# Agricultural Workers’ Perspectives on Stressors, Stress Management Topics and Support Options: A Case Study from the Western U.S.

**DOI:** 10.3390/ijerph22081180

**Published:** 2025-07-25

**Authors:** Grocke-Dewey U. Michelle, Alison Brennan, Brenda J. Freeman, Esmeralda Mandujano, Emma Morano, Doriane Keiser, Don McMoran

**Affiliations:** 1 Department of Human Development and Community Health, Montana State University, Bozeman, MT 59717, USA; alison.brennan@montana.edu (A.B.); emmalee.morano@student.montana.edu (E.M.); doriane.keiser@student.montana.edu (D.K.); 2 Counselor Education Program/Extension, University of Nevada, Reno, NV 89557, USA; brendafreeman@unr.edu; 3 California AgrAbility Program, University of California, Davis, CA 95616, USA; emandujano@ucdavis.edu; 4 Skagit County Extension, Washington State University, Burlington, WA 98233, USA; dmcmoran@wsu.edu

**Keywords:** agriculture, agricultural workers, stress, perceived stress scale, stress management

## Abstract

Agricultural workers—individuals employed for labor in agriculture—are at high risk of various negative health outcomes, with many impacted by both the existence of health disparities and stress. While the issue of farm stress and associated psychosocial health outcomes has been studied in the general agricultural population, research investigating these issues specifically within the agricultural worker population is sparse. This study presents data from the United States Western Region Agricultural Worker Stress Survey (N = 354), which gauged workers’ perceived stress levels, sources of stress, desired stress management topics, and preferred methods of receiving information and support services. Long working hours, working in extreme temperatures, and a lack of time emerged as the top three stressors. On average, workers across the Western region of the U.S. are experiencing a moderate level of stress, with younger workers reporting greater stressor pileup than their older counterparts. Retirement planning was cited as the most preferred stress management topic, regardless of demographic. Lastly, workers chose in-person counseling as the support modality that they would most likely utilize. This research provides a variety of stress management recommendations such as working with farm owners to increase the safety of their operation, investing in face-to-face counseling services, and utilizing community health workers as sources of support.

## 1. Introduction

For the approximate 2.3 million agricultural workers currently employed in the U.S. [1], agricultural work is an arduous experience. It is physically demanding, involves long hours with few breaks, and exposes workers to elements such as intense heat and toxic chemicals. Research highlights that, compared to the general population, agricultural workers are at higher risk of various negative proximal determinants of health such as increased exposure to certain infectious diseases (e.g., different variations of the avian flu [2] and/or COVID-19 [3]), higher rates of occupational injury [4], and elevated risk of sleep deprivation and fatigue [5]. Simultaneously, agricultural workers often experience more negative distal determinants of health such as reduced opportunities for educational achievement [6] and poor housing conditions, which have been reported to be consistently overcrowded and riddled with health hazards, including mold, poor air quality, mildew and water damage [7,8]. These proximal and distal factors often correlate with an increase in heath disparities [9]. Moreover, the combination of these factors, lack of access to care, and health disparities has resulted in the average life expectancy for farmworkers in the U.S., often cited as 49 years, being significantly lower than the national average of 77 years [10].

In addition to these risk factors, there are other contributing factors that not only yield additional adverse health outcomes but have been cited as sources of stress for agricultural workers. The first is a general lack of economic security. The median hourly pay for agricultural workers is approximately USD 16.73 per hour [11], significantly less than the national average wage. Research illustrates that low wages often result in issues such as food insecurity [12,13], limited access to healthcare [14], and lower quality of health care [15]. Even if the wages are not low, the necessity of working in primarily rural areas in and of itself reduces access to healthcare (both physical and mental) when compared with urban workers [16]. Amplified by lack of access to and/or lack of both knowledge and proper training in how to effectively utilize personal protective equipment (PPE), many workers also experience respiratory, dermatological, and other negative health outcomes as a result of pesticide exposure [17].

Numerous recent studies have taken a variety of methodological approaches to investigate various components of the psychosocial health impacts of these agriculture-related stressors. These studies have included an investigation of the key factors impacting farmer mental health in the UK [18], an assessment of the lived experience of high stress and (or) poor mental health in Canadian famers [19], and an analysis of the prevalence and anxiety symptoms among U.S. farm adults and their children [20]. Although more is now known about the complexity of these psychosocial outcomes, it remains a challenge to ascertain which work/life factors are deemed most stressful for agricultural workers, specifically, as many studies combine agricultural producers and workers into one sample. Moreover, recent studies point to the benefit that would come from knowing *how* agricultural workers would prefer to obtain stress management information [21].

Since 1989, the National Agricultural Workers Survey (NAWS) has conducted face-to-face interviews with over 77,000 crop workers across the U.S., collecting demographic, employment, and health data [4]. Although the NAWS contains questions about the stressors that agricultural workers face, it does not query further into either the desired stress management information or the specific ways in which agricultural workers would like to receive such information.

Another factor to consider when assessing the needs of the agricultural worker population is the sheer variability of employment type among agricultural workers. While some work year-round in either a part-time or full-time capacity, others are seasonal workers who are employed for a specific amount of time (a period typically tied to a reoccurring event or cycle such as planting or harvesting) though are not required to be absent from their permanent place of residence. In contrast, migrant farmworkers are seasonal farm workers who travel to their job site and are not reasonably able to return to their permanent residence within the same day [22]. Moreover, approximately 86% of agricultural workers in the U.S. are foreign born, with roughly half lacking legal work authorization [23,24]. For noncitizen agricultural workers numerous other stress-inducing conditions related to their status as foreign workers have been reported such as social isolation, chronic job insecurity and fear of deportation [25,26]. Given that much of the existing literature on stress in agriculture has been conducted with a mix of producers, managers and workers, it becomes difficult to discern the unique stressors that workers face, as well as how their type of agricultural employment may be impacting both their stressors and their stress management preferences.

This project seeks to build upon existing studies by ascertaining the following from a group of agricultural workers in the Western US: (1) their current level of perceived stress, (2) their most common stressors, (3) the types of specific stress management assistance they prefer, and (4) how they would like to receive stress management information and support services. Moreover, this study aims to assess which, if any, demographic variables are related to levels of stress and types of desired stress management assistance. The significance of this study lies in the usefulness of the data in terms of informing programs and strategies to reduce stress in this understudied population.

## 2. Materials and Methods

This study presents data from the U.S. Western Region Agricultural Worker Stress Survey, a tool designed by a team of researchers from across the Western United States who have been working collaboratively on the United States Department of Agriculture (USDA) grant-funded Western Regional Agricultural Stress Assistance Program (WRASAP, for more information see www.farmstress.us, accessed on 1 May 2025.). The present study was the second component of the baseline research portion of a larger project. A separate but similar survey of agricultural producers was conducted prior to this survey of agricultural workers. Findings from the former survey are reported in [27].

### 2.1. Participants

Participants were a convenience and purposive sample of 354 agricultural workers across the Western region of the United States (Alaska, Arizona, California, Colorado, Hawaii, Idaho, Montana, Nevada, New Mexico, Oregon, Utah, Washington, Wyoming) who were at least 18 years old. Participants from California comprised the largest proportion of the sample (*n* = 101, 28.5%), followed by participants from Washington (*n* = 59, 16.7%). Agricultural workers included full-time (*n* = 260, 73.4%) and part-time (*n* = 76, 21.5%) workers; year-round (*n* = 193, 54.5%), seasonal (*n* = 122, 34.5%), and migrant workers *(n* = 22, 6.2%). Most participants (*n* = 285, 80.5%) reported having at least one other job beyond their work on a farm or ranch. Additional participant characteristics are displayed in Table 1.

### 2.2. Procedure

Data collection for this project occurred from March to September 2022. Participants were recruited through each state’s Cooperative Extension Network via email listservs and social media outlets; through local organizations serving agricultural producers (e.g., Farm Bureau, Farm Service Agency); and through the WRASAP network. The survey was administered online using the Qualtrics platform and took approximately 15 min to complete. It was available in English and Spanish, with translation conducted by a Spanish speaking researcher using the form of Spanish spoken by the majority of the Spanish speaking participants. It was subsequently piloted with a group of Spanish-speaking agriculture workers to ensure questions were both culturally appropriate and easy to understand for the target audience, back-translated for accuracy, then finalized. A total of 237 participants (66.9%) completed the survey in English; the Spanish language version was utilized by 117 participants (33.1%).

The survey consisted of 24 questions, including a mix of multiple-choice, matrix, Likert scale, and open-ended items. There were five categories of questions, listed in order of appearance within the survey: sociodemographic variables; perceived stress; stressors; stress management topics; and dissemination strategies (preferred formats/modes of receiving information, and interest in specific outreach projects). Participants who completed the survey were entered in a drawing to receive a USD 50 visa gift card; 10 gift cards were available per state. This research received institutional review board exempt approval at Montana State University (IRB No.: MG020421-EX).

### 2.3. Measures

#### 2.3.1. Demographic Information

##### Age

Participant age in years was a numeric text-entry item. For group-based analysis, we recoded responses into a variable consisting of four age brackets: 18–32, 33–39, 40–48, and 49 and above. We utilized these brackets for consistency across associated lines of research [27].

##### Educational Attainment

Participants indicated “Highest level of education completed” from among eight ordinal options. Due to low frequencies, we collapsed responses to the bottom two options, *2nd/3rd grade* and *8th grade*, into one category (*Less than high school/GED*). See Table 1 for a complete list of levels.

##### Gender

Participants could choose one of three options: *male*, *female*, *non-binary*.

##### Marital Status

Participants indicated their marital status by selecting one of five options: *single, cohabitating, married, divorced, widow/widower.*

##### Race and Ethnicity

Participants were asked, “How would you describe yourself (please select all that apply)”, with eight options given (see Table 1). Participants could specify an unlisted identity using a text-entry item. Responses to the eight options were used to create a variable for group comparison: White non-Hispanic (*n* = 174) and Hispanic/Latinx (*n* = 139). Frequencies for other racial/ethnic identities were too low to be included as groups for comparison.

##### Occupational Information

Participants were asked a series of questions regarding the nature and extent of their involvement in agriculture. Among these questions, they were asked, “How many years have you worked in the agricultural industry (please enter the number of years)?” Single selection items were used to establish *full-time* or *part-time* status, as well as months of the year working on a farm/ranch (12 options). One item measured the type of employment in agriculture in terms of *year-round*, *seasonal*, or *migrant*: “Please indicate whether you are a year-round, seasonal, or migrant farmworker. A seasonal farmworker is a person whose principal employment is in agriculture on a seasonal basis and does not move/travel with the crops. A migrant farmworker establishes a temporary home during the period of employment and travels with the season of the crops to do farm work.” Due to few participants selecting *migrant*, group comparisons included only *year-round* versus *seasonal* workers. Finally, to provide a more complete representation of overall involvement in the workforce, participants were asked, “In addition to working on the farm or ranch, how many additional jobs do you currently hold?” (*0, 1, 2, 3, 4, 5+*).

#### 2.3.2. Perceived Stress

Perceived stress was measured with the validated 10-item version of the Perceived Stress Scale (PSS-10) [28]. The possible range for PSS-10 total scores is 0–40, and total scores can be categorized into three levels, as denoted by the developers: low stress (0–13), moderate stress (14–26), and high stress (27 and higher). Internal consistency reliability was acceptable in the present study, Cronbach’s *α* = 0.76.

#### 2.3.3. Stressors and Stressor Pileup

Participants were prompted to indicate how often, within the last year, each of 35 specific stressors had led them to experience stress. The list of stressors was compiled in consultation with a group of experts in agricultural health and safety. There were four original response options (1 = *never*, 2 = *almost never*, 3 = *fairly often*, and 4 = *very often*) which were collapsed into binary form for analysis (0 = *never* or *almost never*, 1 = *fairly often* or *very often*). Additionally, participants could use a text-entry item to identify specific stressors not included in the list. Consistent with [27], stressor pileup was calculated as a sum of all listed stressors for which a participant selected *fairly often* or *very often*, thus the possible range for stressor pileup in the present study was 0–35. See Table 3 in the Results Section for the list of stressors.

### 2.4. Stress Management Topics

Participants were presented with a list of 23 topics and responded to the prompt, “To help you manage and/or cope with your stress, how interested would you be to learn about the following topics if the content was made available to you?” The four original ordinal response options (1 = *no interest*, 2 = *neutral*, 3 = *interested*, 4 = *very interested*) were collapsed for analysis (0 = *no interest* or *neutral*, 1 = *interested* or *very interested*). If participants selected “Other topic not listed”, they could use a text-entry box to specify additional topics. See Table 4 in the Results Section for the list of stress management topics.

### 2.5. Dissemination Strategies

#### 2.5.1. Preferred Channels/Modes

Participants were presented with 17 potential formats for receiving information. They responded to the prompt, “In thinking about the topics from the previous question that you are interested in learning about, how would you be interested in receiving this information? Select all that apply.” Responses were coded as 1 = selected, 0 = not selected.

#### 2.5.2. Specific Outreach Projects

A set of eight items, constructed to be analogous to planned WRASAP initiatives designed to reduce stress or build coping mechanisms in agricultural workers, described specific types of outreach projects. Participants were asked, “If all were available to you in your community, how likely would you be to make use of the following resources and learning opportunities?” There were four ordinal response options: *very unlikely*, *unlikely*, *likely*, *very likely.* Responses were collapsed for analysis (0 = *very unlikely* or *unlikely;* 1 = *likely* or *very likely*). See Table 5 in the Results Section for the verbatim wording of these items.

### 2.6. Analysis

#### Demographic Analysis

We conducted relevant descriptive analyses (frequencies and percentages, measures of central tendency, measures of variability, etc.) for all variables and examined bivariate associations among the non-categorical demographic variables (age, years in agriculture, months of year in agriculture, number of additional jobs, educational attainment) and stress scores (PSS-10 total score, stressor pileup score). Table 2 displays the correlation matrix for these seven variables. Based on the correlation matrix, we chose to include age and omit educational attainment in subsequent inferential analyses to reduce complexity.

### 2.7. Inferential Analysis

#### 2.7.1. Perceived Stress and Stressor Pileup

We used independent samples *t* tests to assess differences in PSS-10 total score and stressor pileup for demographic variables consisting of two group options (i.e., males vs. females, year-round vs. seasonal, full-time vs. part-time, and White non-Hispanic vs. Hispanic/Latinx). Given the correlations among age, PSS-10 total score, and stressor pileup, we used ANCOVA to examine differences in PSS-10 total score between the four age groups, with stressor pileup as a covariate.

#### 2.7.2. Stress Management Topics and Dissemination Strategies

We conducted a series of chi-square tests for independence to determine whether stress management topic preferences, preferred channels/modes of receiving information, and interest in specific outreach projects were related to gender (*male* or *female*), age group, hours of employment (*part-time* or *full-time*), seasonality of employment (*year-round* or *seasonal/migrant*), and racial/ethnic group (Hispanic/Latinx and White non-Hispanic). Bonferroni corrections for multiple comparisons were used in the interpretation of statistical significance: for stress management topics, *p_i_* = 0.002; for preferred channels/modes of receiving information, *p_i_* = 0.003; and for specific outreach projects, *p_i_* = 0.006.

## 3. Results

### 3.1. Perceived Stress, Specific Stressors and Stressor Pileup

Using the scoring system and category break points delineated by the PSS authors [28], the scores among the sample of agricultural workers indicated a moderate level of stress (*M* = 17.80, *SD* = 6.43). PSS-10 total scores ranged from 0 to 39 within the study sample. Distribution of scores across the three categories was as follows: Low *n* = 79 (22.3%), Moderate *n* = 224 (63.3%), High *n* = 16 (4.5%) (missing *n* = 35, 9.9%), which is comparable to that of agricultural producers in the same region of the U.S. [27].

Long working hours, working in extreme temperatures, lack of time, lack of work/family balance, and low wages emerged as the top five sources of stress for agricultural workers across the region (see Table 3 for complete list of stressors and prevalence; Table S1 displays this information separately for English and Spanish versions of the survey). When asked about other stressors, agricultural workers cited many family/relationship related items such as ‘caring for others’, ‘relationship time’, ‘my family being my coworkers’, ‘addiction of family and neighbors’, and ‘husband that drinks too much’.

Stressor pileup score among the sample of agricultural workers ranged from 0 to 35 (the complete possible range), with a mean of 14.92 (*SD* = 9.76; missing *n* = 26, 7.3%).

### 3.2. Gender Comparisons

Although male and female group means were similar for PSS-10 total score, *M* = 17.91 (*SD* = 6.55) and *M* = 17.51 (*SD* = 6.34) respectively, males reported greater stressor pileup (*M* = 16.45, *SD* = 10.10) than females (*M* = 13.29, *SD* = 9.14), adjusted for heterogeneity of variance, *t*(*df* = 308.6) = 2.93, *p =* 0.004, Cohen’s *d* = 0.327 (small effect).

### 3.3. Seasonality and Hours of Employment Comparisons

There were no differences between year-round and seasonal workers in PSS-10 total score or stressor pileup, and PSS-10 total scores were similar among part-time and full-time workers. However, part-time workers reported lower stressor pileup (*M* = 12.56, *SD* = 7.96) than full-time workers (*M* = 15.88, *SD* = 10.14), corrected for heterogeneity of variance, *t*(*df* = 146.3) = −2.91, *p* = 0.004, Cohen’s *d* = −0.344 (small effect).

### 3.4. Racial/Ethnic Comparisons

PSS-10 total scores were significantly lower among Hispanic/Latinx workers (*M* = 14.46, *SD* = 6.57) than among White, non-Hispanic workers (*M* = 19.46, *SD* = 5.68), corrected for heterogeneity of variance, *t*(*df* = 236.1) = −6.95, *p* < 0.001, Cohen’s *d* = −0.85 (large effect). Similarly, Hispanic/Latinx workers reported substantially lower stressor pileup (*M* = 10.80, *SD* = 8.19) compared to White non-Hispanic workers (*M* = 16.40, *SD* = 9.86), corrected for heterogeneity of variance, *t*(*df* = 282.7) = −5.27, *p* < 0.001, Cohen’s *d* = −0.61 (medium effect).

### 3.5. Age Comparisons

**Figure 1:**
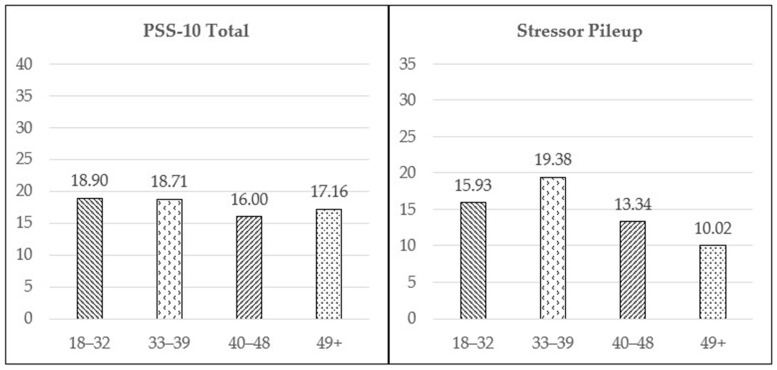
Age group means for stressor variables.

Group means for PSS-10 total score and stressor pileup score are displayed in Figure 1. For reference, one-way ANOVA for stressor pileup score showed a medium effect of age, *F*(3, 309) = 13.09, *p* < 0.001, *eta-squared* = 0.113, and Tamhane’s T2 post hoc comparisons indicated that the youngest age group (18–32) had a higher mean stressor pileup score than the oldest age group (49 and over), *p* < 0.001; and the 33–38 age group had a higher mean stressor pileup score than the 40–48 age group, *p* = 0.002, as well as the oldest age group, *p* < 0.001. One-way ANOVA for PSS-10 total score indicated a small effect of age, *F*(3, 298) = 3.49, *p* = 0.016, *eta-squared* = 0.034, and Tamhane’s T2 post hoc comparisons indicated that the youngest age group had a higher mean PSS-10 total score than the 40–48 age group, *p* = 0.041. There were no differences in PSS-10 total score among age groups in ANCOVA with stressor pileup score as a covariate.

### 3.6. Stress Management Topic Results

The five stress management topics that generated the most interest were retirement planning, financial assistance, physical activity, nutrition and cooking, and sleep. Notably, despite 42.1% of agricultural workers indicating that drugs/alcohol in their community was a stressor (see Table 3), both substance-related stress management topics (alcohol and/or drugs; tobacco/marijuana/vaping cessation) were least desired (refer to Table 4 for a complete list of topics with percentages of participants interested in each; Table S2 displays this information separately for English and Spanish versions of the survey).

### 3.7. Stress Management by Gender

There were no relationships between gender and interest in stress management topics.

### 3.8. Stress Management by Seasonality and Hours of Employment 

There were no relationships between seasonality of employment and interest in stress management topics, nor were there any relationships between hours of employment and interest in stress management topics.

### 3.9. Stress Management by Race/Ethnicity 

There were six topics for which there was a relationship with race/ethnicity, listed from largest to smallest effect size: help learning English (*Χ*^2^_(1)_ = 49.19, *p* < 0.001, *ϕ* = −0.424); training on animal handling (*Χ*^2^_(1)_ = 23.05, *p* < 0.001, *ϕ* = 0.290); sleep (*Χ*^2^_(1)_ = 19.30, *p* < 0.001, *ϕ* = 0.267); help getting kids to school (*Χ*^2^_(1)_ = 14.75, *p* < 0.001, *ϕ* = 0.231); tobacco/marijuana/vaping cessation (*Χ*^2^_(1)_ = 12.12, *p* < 0.001, *ϕ* = 0.210); and retirement planning (*Χ*^2^_(1)_ = 10.39, *p =* 0.001, *ϕ* = 0.193).

For all topics except help learning English, White non-Hispanic workers indicated a higher level of interest. Help learning English was a topic of interest among 70.4% of Hispanic/Latinx workers and 27.7% of White non-Hispanic workers. Training on animal handling was a topic of interest among 50.5% of White non-Hispanic workers and 21.7% of Hispanic/Latinx workers. Help getting kids to school was a topic of interest among 47.2% of White non-Hispanic workers and 24.6% of Hispanic/Latinx workers. Tobacco/marijuana/vaping cessation was a topic of interest among 33.1% of White non-Hispanic workers and 14.7% of Hispanic/Latinx workers. Finally, although retirement planning was a topic of interest among a larger proportion of White non-Hispanic than Hispanic/Latinx workers (70.6% and 51.7%, respectively), it is notable that a majority within both groups were interested in this topic.

### 3.10. Stress Management by Age

There were five topics for which there was a relationship with age, listed from largest to smallest effect size: help getting kids to school (*Χ*^2^_(3)_ = 38.54, *p* < 0.001, *Cramér’s V* = 0.358); parenting (*Χ*^2^_(3)_ = 28.71, *p* < 0.001, *Cramér’s V* = 0.310); tobacco/marijuana/vaping cessation (*Χ*^2^_(3)_ = 24.51, *p* < 0.001, *Cramér’s V* = 0.286); training on animal handling (*Χ*^2^_(3)_ = 20.59, *p* < 0.001, *Cramér’s V* = 0.263); and help getting a driver’s license (*Χ*^2^_(3)_ = 17.62, *p* < 0.001, *Cramér’s V* = 0.242).

Help getting kids to school was a topic of interest for 48.0% of participants ages 18–32, 59.5% of participants ages 33–39, and 35.0% of participants ages 40–48, but only 8.9% of participants ages 49 and above. Relatedly, parenting was a topic of interest for over half of participants ages 18–32 (53.1%) and 33–39 (52.4%), a somewhat smaller proportion of participants ages 40–48 (39.3%), and only 12.5% of participants ages 49 and above.

Although not a popular topic overall, interest in tobacco/marijuana/vaping cessation was higher among younger workers. More than one-third of participants in the younger two age groups were interested in this topic (36.4% of ages 18–32 and 38.1% of ages 33–39), while just 13.1% in the 40–48 age group and 9.1% in the 49 and above age group were interested.

Interest in receiving help getting a driver’s license decreased successively from the youngest age group to the oldest age group (50.0% of ages 18–32, 46.4% of ages 33–39, 29.5% of ages 40–48, and 20.0% of ages 49 and above), with a similar pattern for the topic of training on animal handling (54.5% of ages 18–32, 42.2% of ages 33–39, 30.0% of ages 40–48, and 20.0% of ages 49 and above).

### 3.11. Dissemination Strategy Results

#### 3.11.1. Channels/Modes of Receiving Information

Face-to-face counseling was the most selected mode for receiving information, followed by individual consultation and in-person classes. Notably, telehealth counseling was selected by only 11.8% of participants (refer to Table 5 for a complete list of options and selection frequencies).

#### 3.11.2. Preferred Channels/Modes by Gender

Telehealth counseling was the sole modality for which there was a relationship between selection of the option and gender. More females (18.4%) selected telehealth counseling than males did (3.1%), *Χ*^2^_(1)_ = 22.32, *p* < 0.001, *ϕ* = 0.256.

#### 3.11.3. Preferred Channels/Modes by Seasonality and Hours of Employment 

Although hours of employment (full-time or part-time) were unrelated to any of the channels/modes of receiving information, seasonality of employment was related to two modes of receiving information: podcasts (*Χ*^2^_(1)_ = 13.88, *p* < 0.001, *ϕ* = −0.200) and face-to-face counseling (*Χ*^2^_(1)_ = 12.52, *p* < 0.001, *ϕ* = 0.210). A larger proportion of year-round workers selected podcasts than did seasonal workers (27.5% and 10.7%, respectively). A smaller proportion of year-round workers selected face-to-face counseling than did seasonal workers (24.9% and 45.1%, respectively).

#### 3.11.4. Preferred Channels/Modes by Race/Ethnicity 

Racial/ethnic identity was related to selection for four modalities, listed from largest to smallest effect size: podcasts (*Χ*^2^_(1)_ = 25.37, *p* < 0.001, *ϕ* = 0.285); online library of resources (*Χ*^2^_(1)_ = 22.51, *p* < 0.001, *ϕ* = 0.268); online, self-guided class (no instructor present) (*Χ*^2^_(1)_ = 21.05, *p* < 0.001, *ϕ* = 0.259); and online or webinar class with an instructor (*Χ*^2^_(1)_ = 13.08, *p* < 0.001, *ϕ* = 0.204). In all cases, a larger proportion of White non-Hispanic workers selected the option than did Hispanic/Latinx workers: 31.2% of White non-Hispanic workers selected podcasts, compared to 7.9% of Hispanic/Latinx workers; 31.6% of White non-Hispanic workers selected online library of resources, compared to 9.4% of Hispanic/Latinx workers; 32.8% of White non-Hispanic workers selected online, self-guided class (no instructor present), compared to 10.8% of Hispanic/Latinx workers; and 35.6% of White non-Hispanic workers selected online or webinar class with an instructor, compared to 17.3% of Hispanic/Latinx workers.

#### 3.11.5. Preferred Channels/Modes by Age

There were no relationships between age and selection of channels/modes of receiving information.

### 3.12. Interest in Specific Outreach Projects

Table 5 displays the eight specific outreach projects and overall interest for each one. For all items, more than 50% of respondents indicated interest. The projects that generated the greatest interest—roughly equivalent based on percentages, which account for missing data—were “talking to a peer listener about stress and mental health…”, “learning about stress management and mental health through a brief, self-paced online course”, and “discussing stress, health, and wellness topics with someone you know well at informal events…”. “Participating in virtual (e.g., Zoom, Skype) informal discussion groups” generated the lowest interest. Results of demographic-focused chi-square analysis are detailed below.

#### 3.12.1. Specific Outreach Projects by Gender 

There were no relationships between gender and interest in specific outreach projects.

#### 3.12.2. Specific Outreach Projects by Seasonality and Hours of Employment 

Although hours of employment were unrelated to interest in specific outreach projects, there was a relationship between seasonality of employment and interest for one specific outreach project, “Discussing stress, health, and wellness topics with a representative working on behalf of your community or health organization”, *Χ*^2^_(1)_ = 10.43, *p =* 0.001, *ϕ* = 0.195. More seasonal workers (78.4%) than year-round workers (59.8%) were interested in this outreach project.

#### 3.12.3. Specific Outreach Projects by Race/Ethnicity

There was a relationship between racial/ethnic identity and interest for one specific outreach project, “Participating in virtual (e.g., Zoom, Skype) informal discussion groups”, *Χ*^2^_(1)_ = 17.72, *p* < 0.001, *ϕ* = 0.256. A larger proportion of White non-Hispanic workers (69.0%) than Hispanic/Latinx workers (43.5%) were interested in this outreach project.

#### 3.12.4. Specific Outreach Projects by Age

There was a relationship between age and interest for two specific outreach projects: “Participating in online or telephone counseling/therapy” (*Χ*^2^_(3)_ = 28.55, *p =* 0.011, *Cramér’s V* = 0.313); and “Participating in a support group” (*Χ*^2^_(3)_ = 13.32, *p* = 0.004, *Cramér’s V* = 0.212). In both cases, more than half of workers within all *except* the oldest age group were interested in the outreach project: 57.1% of ages 18–32, 84.1% of ages 33–39, and 60.0% of ages 40–48 were interested in online or telephone counseling/therapy, compared to 40.4% of ages 49 and over; 68.7% of ages 18–32, 73.5% of ages 33–39, and 61.7% of ages 40–48 were interested in support groups, compared to 44.4% of ages 49 and over.

## 4. Discussion

### 4.1. Discussion of Perceived Stress, Stressor Pileup and Specific Stressors

Based on the average PSS score, the results suggest that most agricultural workers in this Western regional sample of the United States are experiencing a moderate level of stress (mean PSS score = 17.8). This score indicates that stress may be impacting workers’ daily activities, and that taking steps to help positively cope with stress could help to prevent future physical, psychological, and emotional consequences.

The research also identified a pattern between age and stressor pileup. Compared to the oldest age group, the two younger age groups (18–32 and 33–39 years of age) reported greater stressor pileup. Furthermore, when controlling for stressor pileup, there are no differences in PSS total among any age group. This relationship could be explained by socioemotional selectivity theory, which highlights that as people start to perceive their time in life as limited, how they prioritize both tasks and their emotions can potentially lend itself to better stress management through selective social engagement and a focus on the positive [29]. The data suggests targeting stress management and coping resources to the two younger age groups. This is particularly important given that research indicates that the capacity to cope with stress is a predictor of life expectancy as well as life satisfaction [30].

Another demographic difference in the PSS and stressor pileup data was between White, non-Hispanics and Hispanic/Latinx workers. Hispanic/Latinx workers reported lower PSS scores (Hispanic/Latinx mean PSS score = 14.5, White non-Hispanic mean PSS score = 19.6) as well as lower stressor pileup scores (Hispanic/Latinx mean pileup score = 10.9, White, non-Hispanic mean pile up score = 16.5). These differences could be in part explained by cultural components such as the support of families (familism), the belief that events are predetermined and therefore individuals do not have control over their situation (fatalism) and leaving things in God’s hands (religiosity), as all have been documented to support mental health resilience in Hispanics [31,32,33]. Hispanic culture is also thought to be a collectivistic culture. Members of collectivistic cultures often value relationships and community engagement, a value which could help to decrease social isolation, a key factor in suicide risk [34].

Notably, there was no statistically significant difference in either PSS or stressor pileup based on seasonality of agricultural employment (year-round vs. seasonal). This indicates that type of employment may not play as much of a role in stress levels as other demographic variables.

In terms of which stressors are most predominant in agricultural workers’ lives, this research found that long working hours, working in extreme temperatures, lack of time, lack of work/family balance, and low wages were the five most cited. Some of these ‘working condition-related’ stressors could be mitigated by employers’ efforts such as providing access to water, shade and rest, as well as by providing trainings to workers to prevent and recognize heat related illnesses (for example, the National Center for Farmworker Health has a recorded online training [35]). Various farm safety publications also emphasize the importance of not letting new workers work alone, as almost half of all heat-related deaths occur on a worker’s very first day on the job [36,37]. Moreover, this statistic points to the importance of gradually introducing workers to extreme temperature conditions to facilitate heat tolerance. Others point to utilizing a heat app for Andriod and IPhone that uses the heat index as a screening tool [38], which sounds feasible considering that the most recent NAWS report cited that 99% of crop workers reported having digital access [39].

Incorporating some flexibility in agricultural workers’ schedules could also help to mitigate stressors such as ‘long working hours’ and ‘lack of time’, whereby also helping to improve workers’ ‘work/family balance’. In fact, ‘flexible hours’ was cited as the top employment perk, according to the 2022 Agricultural Candidate and Employee Benefits Survey Report [40]. In the case of a farm operation where telework or remote work is likely impossible, employers should consider flexibility in time and/or days off. A multitude of research has reported on the health benefits associated with flexible work hours such as reduced stress, fewer stress-related illnesses, lower absenteeism, and improved relationships (e.g., more time spent with children, increased marital satisfaction) [41]. These positive outcomes help to explain the positive association between flexible work hours and increased job productivity—if workers have some level of flexibility, they are often more motivated at work and report an increased sense of job satisfaction. Seasonal workers are often unemployed during some months of the year (or they accept non-agricultural jobs). The results may mask information about the season where long work hours are problematic, as a typical direct worker will work very long hours during harvest, but may be unemployed in the winter [36]. Notably, these cited stressors are for the most part outside of the control of the individual. As control itself is a key stress management strategy, this may aid in explaining why these particular stressors were more important to participants [38].

### 4.2. Discussion of Stress Management Topics and Dissemination Strategies

Retirement planning was the stress management topic that agricultural workers, regardless of age, were most interested in learning about. This speaks to their desire to better understand—and be in control over—their own personal financial situation. This finding is supported by research highlighting the importance of tailoring retirement financial planning to age groups, occupation, and ethnicity [42].

Data also illustrates that younger agricultural workers are far more interested in learning about a much higher variety of stress management topics than those in older age groups. While this could also tie back to socioemotional selectivity theory as older individuals may not want to invest time into something new, this finding again supports the need for stress management programs that target younger workers. Although on average, tobacco/marijuana/vaping cessation was the least desired stress management topic, the younger two age groups had far greater interest in learning about tobacco/marijuana/vaping cessation information as a stress management strategy.

In contrast to the high frequency of responses to the question regarding stress management topics (see frequencies in Table 4), participants responded with relatively low frequency in terms of which channels/modes of receiving information they would utilize (see frequencies in Table 5). Even so, data points to agricultural workers far preferring face-to-face counseling over telehealth counseling, which provides interesting insight, especially considering recent research that illustrates both the clinical effectiveness [43] and cost effectiveness [44] of telehealth counseling. While numerous ongoing efforts exist to provide free telehealth counseling services to members of the agricultural community, this finding begs the question of whether unaddressed acceptability variables (high stigma; discomfort with technology) remain barriers to seeking such support.

Our data illustrate that in-person counseling is much needed in rural areas, where providers can offer counseling in both English and Spanish, and are preferably trained in the culture of agriculture. Specifically for non-White Hispanic workers, mental health support via a primary care physician would be a good starting point, as evidence shows that Latinos are more likely to seek help for a mental health issue from a primary care provider as opposed to a mental health specialist [33]. Moreover, finding a way to subsidize counseling for this population would seem appropriate, as, according to the most recent findings from the NAWS, only 52% of workers responded that they themselves had health insurance [39].

When asked about specific ongoing outreach projects participants would be interested in, a high percentage (72.2%) indicated that talking to a peer listener about stress and mental health (see Table 5) would be of interest. These findings support programs that utilize farmer-to-farmer community programming as well as community health workers (CHW), as CHWs are uniquely positioned to help reduce adverse health outcomes, and thereby stress, in the agriculture worker population [45,46]. Moreover, there was a significantly higher difference in interest in CHWs among seasonal workers with 74.8% expressing interest.

### 4.3. Limitations

This research is not without limitations. For one, in every state other than California, this survey was largely disseminated online. The research team simply did not have the capacity or human capital to disseminate in-person, other than in California where the research team had more resources. This could have yielded a selection bias. Second, the survey questions were created via an iterative process that involved a community advisory board. However, some of the questions (including the question that asked participants about their stressors) had a pre-determined list of occupational-related stressors that participants could choose from. The research team could have unintentionally missed stressors that participants are impacted by, such as familial and caring-related stressors that women are more likely to face. This omission could have contributed to females reporting lower stressor pileup. Another limitation to note is that given the geographic area covered, the sample size was small. Although a small sample may not have robust representation, the data still carry meaning and value in representing voices from the agricultural worker community, one that is underrepresented in the literature. Unfortunately, due to some states having a small sample, we were not able to make any state-based comparisons. The survey was also distributed between the months of March–September, which could have impacted both the amount and types of stress that workers were facing at that specific time. Lastly, it could have been that workers were reluctant to identify as migrant workers, as they may have had concerns about their citizenship status, etc. Given this potential, and the low participation of those that did identify as migrant workers, we chose to not report on unique stressors or desired topics of stress management for the subpopulation of migrant agricultural workers.

## 5. Conclusions

This research provides insight into factors causing stress for segments of the agricultural worker population in the Western U.S., stress management topics workers are most interested in learning more about, and the information and support options they would prefer. We encourage individuals and organizations working to improve the health outcomes of the agricultural worker community to tailor their programming and method of delivery based on agricultural workers’ stated preferences.

Most of the cited stressors that agricultural workers face—long working hours, working in extreme temperatures, lack of time, lack of work/family balance, and low wages—although largely out of the control of the worker, can be mitigated or attenuated by the producer and/or farm/ranch owner. As previously mentioned, some effective strategies to consider are better managing work conditions and, when possible, providing more flexibility in workers’ schedules. In terms of which stress management topics workers wanted to learn about, it is encouraging that workers, in general, are interested in a variety of stress management topics to help them cope and/or reduce their stress. These findings highlight that there are many entry points for existing organizations and programs to support agricultural workers. In terms of the best way to reach agricultural workers with stress management information, our research highlights that more efforts should be made to provide accessible, subsidized, face-to-face counseling to the agricultural worker community, as this was their most preferred option. Investing in CHWs to share health promotion and stress management information on a local level is also recommended.

Though the study of stressors is by its nature deficit-based, we end with a note on resiliency. One significant resiliency factor for farm and ranch workers may be the opportunity to work outside. Research on mental health and horticulture indicates that working outdoors in a natural environment is an important stress-protective factor [47,48]. Another important resiliency issue is that farm workers, unlike farm producers, reportedly experience a peer-to-peer comradery as they work side-by-side with other workers. Teamwork is often essential, and has been found to boost worker morale and lead to pride in the work performed together [49]. We encourage those working to support the agricultural worker population to capitalize on these resiliency factors when creating and/or implementing mental wellness supports.

To conclude, given the indisputable link between high stress and increased mortality [50], any work to lower stress levels among the agricultural community will not only yield short-term improvements in well-being and life satisfaction but will help this segment of the population live longer, healthier lives. Given the changing U.S. farm worker demographics—e.g., women are an increasing share of the hired farm workforce, up to 22.6% in 2022 [51]—current research that assesses how stressors and stress management preferences vary by demographic group needs to be readily available. We encourage those working to improve the lives of agricultural workers in the Western U.S. to ensure that their health education, promotion programs, and interventions are tailored to fit the needs and wants of each group comprising the agricultural worker population. Further research should assess the effectiveness of targeted stress management outreach programs in order to elucidate both successes and challenges. Moreover, similar research should be conducted in other parts of the world where farm worker stress is high, and life expectancy is lower than that of the general population as a means to illuminate how best to support this portion of the global agricultural economy.

## Figures and Tables

**Table 1:** Participant characteristics (N = 354).

Characteristic	*n*	% ^1^
Gender		
Male	194	54.8
Female	147	41.5
Non-binary	1	0.3
Marital Status		
Married	219	61.9
Single	60	16.9
Cohabitating	41	11.6
Divorced	20	5.6
Widow/er	2	0.6
Educational Attainment		
Less than high school diploma	71	20.0
High school diploma/GED	112	31.6
Trade school/associate’s degree	20	5.6
Some college	53	15.0
Bachelor’s degree	58	16.4
Master’s degree	22	6.2
Doctoral degree/terminal degree	4	1.1
Race/Ethnicity		
White or European American	175	49.4
Hispanic or Latino(a)	139	39.3
American Indian or Alaska Native	14	4.0
Asian or Asian American	13	3.7
Black or African American	5	1.4
Native Hawaiian or Pacific Islander	4	1.1
Middle Eastern or North African	1	0.3
Multi-ethnic	1	0.3
Number of additional jobs		
0	54	15.3
1	190	53.7
2	75	21.2
3	9	2.5
4	4	1.1
5+	7	2.0
	Mean	SD
Age (*n* = 335)	38.81	11.93
Years working in agriculture (*n* = 326)	14.26	11.96
Months of year working in agriculture (*n* = 324)	8.75	3.28

^1^ Percentages do not total 100 due to missing data. Demographic variable missing data ranged from *n* = 12 (3.4%) to *n* = 15 (4.2%). With missing included (*n* = 12, 3.4%), Race/Ethnicity exceeds 100 due to multiple selections (select all that apply).

**Table 2:** Correlations.

Variable	1	2	3	4	5	6
1. Age	−					
2. Years in agriculture	0.705 ***	−				
3. Months of year in agriculture	0.202 ***	0.308 ***	−			
4. Additional jobs	−0.104	0.012	−0.086	−		
5. Educational attainment	−0.150 **	−0.076	−0.047	0.134 *	−	
6. Stressor pileup	−0.226 ***	−0.267 ***	−0.195 ***	0.174 **	0.044	−
7. PSS-10 total score	−0.147 *	−0.211 ***	−0.058	0.164 **	0.246 ***	0.499 ***

* *p* < 0.05, ** *p* < 0.01, *** *p* < 0.001.

**Table 3:** Stressor prevalence.

Stressor	*n* (%) ^1^
Long working hours	227 (71.8)
Working in extreme temperatures	216 (70.8)
Lack of time (no time to rest, complete tasks well, etc.)	202 (64.5)
Lack of work/family balance	201 (64.2)
Low wages	188 (63.7)
Financial worries (loans, debts, bank pressure etc.)	187 (59.9)
Technology issues	173 (55.8)
Language barrier	145 (49.8)
Illness/injury preventing ability to work	148 (47.6)
Poor workplace communication	147 (47.0)
Insecure job status	144 (46.8)
Exposure to pesticides or chemicals	144 (46.0)
Mobile lifestyle	136 (45.5)
Social isolation	143 (45.0)
Crop/plant disease	140 (44.9)
COVID-19	138 (44.8)
Lack of entertainment	139 (44.3)
Lack of access to health insurance	130 (44.2)
Cognitive/emotional disability	136 (43.6)
Livestock issues	133 (43.5)
Drug and alcohol use in the community	134 (42.1)
Grief (death of a loved one or community member)	123 (42.1)
Family separation	121 (41.6)
Wildfire smoke conditions	124 (40.7)
Discrimination/racism	128 (40.4)
Lack of access to medical care	120 (40.1)
Transportation	125 (39.3)
Physical isolation	121 (38.3)
Pesticide safety regulation violations	107 (35.5)
Substandard housing	106 (35.5)
Difficulty getting to U.S. for work	99 (34.9)
Lack of water for drinking and hygiene	101 (32.9)
Lack of employer provided PPE	96 (31.3)
Physical disability	91 (31.0)
Community violence	82 (23.2)

^1^ Number and percentage of respondents who selected “*often*” or “*very often*”. Missing *n* excluded from denominator for percentages. Missing ranged from *n* = 35 (9%) to *n* = 70 (19.8%).

**Table 4:** Interest in stress management topics.

Topic	*n* (%) ^1^
Retirement planning	192 (62.5)
Financial assistance	189 (61.8)
Physical activity	186 (60.6)
Nutrition and cooking	178 (57.8)
Sleep	169 (55.2)
Relationship support	166 (53.9)
Career/vocational support	157 (51.1)
Training on tractor/equipment driving or safety	155 (51.0)
Mindfulness	153 (50.5)
Support groups	154 (50.3)
Mental health counseling	152 (49.5)
Help learning to speak English	149 (49.0)
Gardening	144 (47.5)
Physical rehabilitation	144 (46.8)
Training on pesticide safety	138 (45.2)
Parenting	132 (43.3)
Grief counseling	127 (41.6)
Help getting your kids to school	127 (41.4)
Training on animal handling	122 (40.1)
Help getting a driver’s license	120 (39.1)
Alcohol and/or drugs	96 (31.1)
Tobacco/marijuana/vaping cessation	82 (26.8)

^1^ Number and percentage of respondents who selected “*interested*” or “*very interested*”. Missing *n* excluded from denominator for percentages. Missing ranged from *n* = 45 (12.7%) to *n* = 51 (14.4%).

**Table 5:** Dissemination strategies.

Item	*n* (%) ^1^
Preferred Channels/Modes	
Face-to-face counseling	116 (39.2)
Individual consultation	96 (32.4)
In-person class in your community	92 (31.1)
Online or webinar class with an instructor	92 (31.1)
Online, self-guided class on your own time (no instructor present)	84 (28.4)
Social media (e.g., Facebook)	82 (27.7)
Online library of resources	77 (26.0)
General telephone help line	73 (24.7)
Podcast	72 (24.3)
Printed resources mailed to you	71 (24.0)
Radio	54 (18.2)
TV	54 (18.2)
Ag-specific telephone help line (e.g., Farm Aid)	50 (16.9)
Printed resources available at your local Extension office	42 (14.2)
Religious/church/spiritual leaders	35 (11.8)
Telehealth counseling	35 (11.8)
Printed resources available at grocery stores, gas stations, or other local stores	32 (10.8)
Specific Outreach Projects	
Talking to a peer listener about stress and mental health (a peer listener is a member of the agricultural community who is trained to listen and respond to their neighbors and direct them to available resources)	216 (72.2)
Learning about stress management and mental health through a brief, self-paced online course	217 (72.1)
Discussing stress, health, and wellness topics with someone you know well at informal events (during a potluck, at a backyard barbecue, at a coffee shop)	218 (71.9)
You or someone from your household/family participating in community planning sessions to identify and address health and wellness issues in your community	202 (66.9)
Discussing stress, health, and wellness topics with a representative working on behalf of your community or health organization	201 (66.8)
Participating in a support group	196 (64.7)
Participating in online or telephone counseling/therapy	186 (62.2)
Participating in virtual (e.g., Zoom, Skype) informal discussion groups	176 (58.9)

^1^ Organized in descending order based on percentages. Preferred Channels/Modes: number and percentage of respondents who selected each option; percentages calculated using completion *n* as denominator; missing *n* = 58 (16.4%) for item set. Specific Outreach Projects: number and percentage of respondents who selected “*likely*” or “*very likely*”; percentages calculated using completion *n* as denominator; missing ranged from *n* = 51 (14.4%) to *n* = 55 (15.5%).

## Data Availability

Anonymized data may be available upon reasonable request but would need to be confirmed via the ethics board.

## Supplementary Materials

The following supporting information can be downloaded at: https://www.mdpi.com/article/10.3390/ijerph22081180/s1, Table S1: Stressor prevalence displayed separately by survey version; Table S2: Interest in stress management topics displayed separately by survey version.

## Abbreviations

The following abbreviations are used in this manuscript:

NAWS	National Agricultural Workers Survey
USDA	United States Department of Agriculture
WRASAP	Western Regional Agricultural Stress Assistance Program
PSS	Perceived Stress Scale
OSHA	Occupational Safety and Health Administration
CHW	Community Health Workers
