# Supplementary material for: Agricultural Workers’ Perspectives on Stressors, Stress Management Topics and Support Options: A Case Study from the Western U.S
Source: Int J Environ Res Public Health. 2025 Jul 25;22(8):1180. doi: 10.3390/ijerph22081180 (PMC12386628; doi:10.3390/ijerph22081180)
Supplement: Supplementary file 1 [file ijerph-22-01180-s001.zip › ijerph-3755330-supplementary.pdf]

Supplementary Materials

Table S1. Stressor prevalence displayed separately by survey version.

Stressor	English (*n* = 237) *n (%)*	Spanish (*n* = 117) *n (%)*
Long working hours	167 (75.6)	60 (63.2)
Working in extreme temperatures	164 (75.6)	52 (59.1)
Lack of time (no time to rest, complete tasks well, etc.)	156 (71.6)	46 (48.4)
Lack of work/family balance	152 (70.7)	49 (50.0)
Low wages	142 (68.9)	46 (51.7)
Financial worries (loans, debts, bank pressure, etc.)	149 (68.0)	38 (40.9)
Technology issues	141 (65.3)	32 (34.0)
Language barrier	91 (44.6)	54 (62.1)
Illness/injury preventing ability to work	116 (53.2)	32 (34.4)
Poor workplace communication	118 (54.1)	29 (30.5)
Insecure job status	111 (51.2)	33 (36.3)
Exposure to pesticides or chemicals	107 (49.5)	37 (38.1)
Mobile lifestyle	108 (52.7)	28 (29.8)
Social isolation	114 (53.0)	29 (28.2)
Crop/plant disease	122 (57.5)	18 (18.0)
COVID-19	97 (44.7)	41 (45.1)
Lack of entertainment	112 (52.1)	27 (27.3)
Lack of access to health insurance	100 (47.8)	30 (35.3)
Cognitive/emotional disability	113 (52.8)	23 (23.5)
Livestock issues	123 (58.9)	10 (10.3)
Drug and alcohol use in the community	106 (49.3)	28 (27.2)
Grief (death of a loved one or community member)	77 (39.3)	46 (47.9)
Family separation	83 (42.6)	38 (39.6)
Wildfire smoke conditions	105 (51.2)	19 (19.0)
Discrimination/racism	99 (41.8)	29 (28.2)
Lack of access to medical care	95 (44.2)	25 (29.8)
Transportation	106 (48.0)	19 (19.6)
Physical isolation	99 (46.0)	22 (21.8)
Pesticide safety regulation violations	88 (43.6)	19 (19.2)
Substandard housing	88 (43.6)	18 (18.6)
Difficulty getting to U.S. for work	83 (43.2)	16 (17.4)
Lack of water for drinking and hygiene	92 (44.0)	9 (9.2)
Lack of employer provided PPE	80 (38.3)	16 (16.3)
Physical disability	71 (35.5)	20 (21.3)
Community violence	71 (32.9)	11 (10.7)

Number and percentage of respondents who selected *"often"* or *"very often"*. Missing *n* excluded from denominator for percentages.

Table S2. Interest in stress management topics displayed separately by survey version.

Topic	English (*n* = 237) *n (%)*	Spanish (*n* = 117) *n (%)*
Retirement planning	144 (69.6)	48 (48.0)
Financial assistance	131 (63.3)	58 (58.6)
Physical activity	134 (64.7)	52 (52.0)
Nutrition and cooking	121 (58.2)	57 (57.0)
Sleep	134 (64.7)	35 (35.4)
Relationship support	126 (60.6)	40 (40.0)
Career/vocational support	110 (53.1)	47 (47.0)
Training on tractor/equipment driving or safety	120 (58.3)	35 (35.7)
Mindfulness	102 (50.0)	51 (51.5)

Support groups	113 (54.6)	41 (41.4)
Mental health counseling	109 (52.7)	43 (43.0)
Help learning to speak English	77 (37.2)	72 (74.2)
Gardening	103 (50.7)	41 (41.0)
Physical rehabilitation	110 (52.9)	34 (34.0)
Training on pesticide safety	104 (50.5)	34 (34.3)
Parenting	100 (48.5)	32 (32.3)
Grief counseling	85 (41.3)	42 (42.4)
Help getting your kids to school	106 (51.2)	21 (21.0)
Training on animal handling	100 (48.5)	22 (22.4)
Help getting a driver's license	86 (41.3)	34 (34.3)
Alcohol and/or drugs	70 (33.7)	26 (25.7)
Tobacco/marijuana/vaping cessation	69 (33.2)	13 (13.3)

Number and percentage of respondents who selected *"interested"* or *"very interested"*. Missing *n* excluded from denominator for percentages.
